# Factors Associated With COVID-19 Death in the United States: Cohort Study

**DOI:** 10.2196/29343

**Published:** 2022-05-12

**Authors:** Uan-I Chen, Hua Xu, Trudy Millard Krause, Raymond Greenberg, Xiao Dong, Xiaoqian Jiang

**Affiliations:** 1 School of Biomedical Informatics The University of Texas Health Science Center at Houston Houston, TX United States; 2 Department of Management, Policy, and Community Heath School of Public Health The University of Texas Health Science Center at Houston Houston, TX United States; 3 Department of Population and Data Sciences Peter O'Donnell School of Public Health University of Texas Southwestern Medical Center Dallas, TX United States; 4 Department of Epidemiology, Human Genetics, and Environmental Health School of Public Health The University of Texas Health Science Center at Houston Houston, TX United States

**Keywords:** COVID-19, risk factors, survival analysis, cohort studies, EHR data

## Abstract

**Background:**

Since the initial COVID-19 cases were identified in the United States in February 2020, the United States has experienced a high incidence of the disease. Understanding the risk factors for severe outcomes identifies the most vulnerable populations and helps in decision-making.

**Objective:**

This study aims to assess the factors associated with COVID-19–related deaths from a large, national, individual-level data set.

**Methods:**

A cohort study was conducted using data from the Optum de-identified COVID-19 electronic health record (EHR) data set; 1,271,033 adult participants were observed from February 1, 2020, to August 31, 2020, until their deaths due to COVID-19, deaths due to other reasons, or the end of the study. Cox proportional hazards models were constructed to evaluate the risks for each patient characteristic.

**Results:**

A total of 1,271,033 participants (age: mean 52.6, SD 17.9 years; male: 507,574/1,271,033, 39.93%) were included in the study, and 3315 (0.26%) deaths were attributed to COVID-19. Factors associated with COVID-19–related death included older age (≥80 vs 50-59 years old: hazard ratio [HR] 13.28, 95% CI 11.46-15.39), male sex (HR 1.68, 95% CI 1.57-1.80), obesity (BMI ≥40 vs <30 kg/m_2_: HR 1.71, 95% CI 1.50-1.96), race (Hispanic White, African American, Asian vs non-Hispanic White: HR 2.46, 95% CI 2.01-3.02; HR 2.27, 95% CI 2.06-2.50; HR 2.06, 95% CI 1.65-2.57), region (South, Northeast, Midwest vs West: HR 1.62, 95% CI 1.33-1.98; HR 2.50, 95% CI 2.06-3.03; HR 1.35, 95% CI 1.11-1.64), chronic respiratory disease (HR 1.21, 95% CI 1.12-1.32), cardiac disease (HR 1.10, 95% CI 1.01-1.19), diabetes (HR 1.92, 95% CI 1.75-2.10), recent diagnosis of lung cancer (HR 1.70, 95% CI 1.14-2.55), severely reduced kidney function (HR 1.92, 95% CI 1.69-2.19), stroke or dementia (HR 1.25, 95% CI 1.15-1.36), other neurological diseases (HR 1.77, 95% CI 1.59-1.98), organ transplant (HR 1.35, 95% CI 1.09-1.67), and other immunosuppressive conditions (HR 1.21, 95% CI 1.01-1.46).

**Conclusions:**

This is one of the largest national cohort studies in the United States; we identified several patient characteristics associated with COVID-19–related deaths, and the results can serve as the basis for policy making. The study also offered directions for future studies, including the effect of other socioeconomic factors on the increased risk for minority groups.

## Introduction

The COVID-19 pandemic has brought an unprecedented crisis in global public health since it first appeared in late 2019. By the end of 2020, 19,943,605 confirmed cases and 344,497 deaths were reported in the United States [1], which was the largest number of any country in the world. Although based on a short observation period or within a single geographical region, reports and studies from the early stage of the pandemic revealed high hospitalization and mortality rates [2-4]. Identifying prognostic factors can help determine patients at the highest risk for poor outcomes and focus interventions accordingly.

Many studies have been conducted on this topic in the United States. For example, a study of 2215 adult COVID-19 patients admitted to intensive care units showed that patients older than 80 years had a much higher risk of COVID-19–related death. Men also were at an increased risk [5]. Similar findings also were reported from a study involving 64,781 patients treated in 592 US hospitals during April and May 2020 [6]. Pre-existing conditions including obesity, coronary artery disease, cancer, liver or kidney dysfunction, neurological disorder, diabetes, and dementia were each associated with raised risks of a severe outcome [5,6]. Although many studies have explored the linkage between patient characteristics and COVID-19 death, most of them involved limited sample sizes and relatively short time spans. Recently, a large cohort study of 1,926,526 patients with 174,568 COVID-19 confirmed cases from the National COVID Cohort Collaborative (N3C), a centralized national data resource, reported that age, male sex, liver disease, dementia, African American and Asian race, and obesity were associated with poor outcomes [7]. There is a scarcity of studies using large national data similar to the N3C cohort in the United States to provide accurate and reliable findings.

In 2020, a UK team used National Health Service data to build the OpenSAFELY platform and to conduct a cohort study of 17 million people to investigate factors associated with COVID-19–related deaths in England [8]. The findings showed that people over 80 years old had a 20 times higher risk compared with those aged 50-59 years (hazard ratio [HR] 20.6, 95% CI 18.7-22.68). Men had slightly higher risk than women (HR 1.59, 95% CI 1.53-1.65). Minority groups, including mixed-race, South Asian, and Black people, were at higher risks than White people. In addition, obesity and most comorbidities, including cardiac, pulmonary, kidney disease, and malignancies, were all associated with higher risks of COVID-19–related deaths.

To understand whether these factors proposed in the OpenSAFELY study, including age, sex, and other comorbidities, were also linked to higher risks of COVID-19–related deaths among the US population during the similar time window, we conducted a study that expands upon the UK study through the analysis of the Optum de-identified COVID-19 electronic health record (EHR) data set and compared findings with the aforementioned N3C cohort with over 1 million patient records between February 1, 2020, and August 31, 2020, in the United States.

## Methods

### Study Design

This study was designed to replicate the UK OpenSAFELY [8] study within the constraints of the available data. We conducted a cohort study using data from the Optum de-identified COVID-19 EHR data set. The study started on February 1, 2020, which was the earliest date Optum began compiling the COVID-19 data. The date was 10 days after the first COVID-19 confirmed case and several weeks before the first reported COVID-19–related death in the United States. The study ended on August 31, 2020, which was the latest accessible record released by Optum by the time the study was performed. In the primary analysis, all eligible participants were included in the study regardless of their SARS-CoV-2 test results (the full cohort) to assess risks among the general population. For the analysis among COVID-19 patients, a subset of patients was extracted from the full cohort with at least one lab-confirmed polymerase chain reaction (PCR)–positive SARS-CoV-2 test result or with diagnosis code U07.1 or B97.29 between February 1, 2020, and August 17, 2020 (the date was chosen 2 weeks before the study ended to allow the outcome to fully develop). No randomization was conducted. No investigator was involved in the outcome assessment.

### Data Source

The Optum COVID-19 data set was provided by Optum to the University of Texas (UT) Center for Health Care Data, University of Texas Health Science Center (UTHealth) School of Public Health, and UTHealth School of Biomedical Informatics (SBMI) Data Service. The data set accessed throughout the study was locally hosted by SBMI. It comprised longitudinal EHR data derived from a network of health care provider organizations across the United States. The data were certified as de-identified by an independent statistical expert following Health Insurance Portability and Accountability Act statistical de-identification rules and managed according to Optum customer data use agreements. Clinical and other medical administrative data were obtained from both inpatient and ambulatory EHRs, practice management systems, and numerous other internal systems. Information was processed, normalized, and standardized across the continuum of care from both acute inpatient stays and outpatient visits. Optum data elements included demographics, medications prescribed and administered, lab results, vital signs, other observable measurements, clinical and inpatient stay administrative data, and coded diagnoses and procedures.

All authors were authorized to access the Optum COVID-19 data set and were compliant with the data use agreements.

### Study Population and Observation Period

Considering many of the risk factors investigated in this study are chronic health conditions that are more commonly present in adult patients, the study population included only adult men and women aged 18 years or older on or before February 1, 2020. To be included in the study, participants must have had at least 1 year of prior observation before the study start date in order to adequately capture their baseline characteristics. Participants also were required to have EHRs for the prior year to be considered eligible for inclusion. In addition, participants with missing demographics, including sex, age, and region, were excluded from the study. Eligible participants were followed from February 1, 2020, until their deaths due to COVID-19, deaths due to other causes, or the end of the study (August 31, 2020). The Optum data set used in this study was delivered on September 3, 2020, which contained some death data for early September. However, we elected to end the study period a few days earlier to account for possible delays in data delivery.

### Ethical Considerations

Data for this study were provided by Optum and remain on the servers of the Biomedical Informatics Group-the Analytics Research Center, SBMI, UTHealth. No individually identifiable information was provided, and no participants were contacted by the investigators directly. The secondary analysis of this de-identified data was approved by the Committee for the Protection of Human Subjects, University of Texas Health Science Center at Houston (the UTHSC-H institutional review board) under protocol HSC-SBMI-20-1194.

### Outcome

The outcome of interest was COVID-19–related deaths. All non-COVID-19–related deaths or surviving patients were censored at the time of death or the end of the study, respectively. Due to the de-identification of data, only the death year and month were available. Neither the exact death day nor the cause of death was provided. We, therefore, used an indirect way to define COVID-19–related deaths: If the month of a patient’s last COVID-19 diagnosis (International Classification of Diseases [ICD]-10 codes U07.1 on or after February 1, 2020, or ICD-10 codes B97.29 on or after February 20, 2020) matched or was within 1 month after the death month and any of the other most recent recorded dates (hospital discharge date, health service encounter date, diagnosis date, lab test ordered date, prescription date, and medical procedure date) was the same as or within 1 month after the death month, the patient was considered to have experienced a COVID-19–related death. The extra 1-month window was included to account for the possible delay of data entry. For example, if a patient had a positive COVID-19 diagnosis on April 4 and died in April, with any of the aforementioned dates falling in April, the patient was considered to have died from COVID-19. However, if the patient had a positive diagnosis in February but died in April, the patient would be considered to have died from other causes. To determine the death day, we defined the most recent recorded date among the aforementioned dates that matched the death month as the presumptive date of death. For those without matching records on the death month, the presumptive date of death was set to the 15th of the death month as the midpoint of possible death dates.

### Covariates

Potential risk factors and their categorizations in this study generally followed those used in the OpenSAFELY study [8]. Age was grouped into 6 categories: 18-39, 40-49, 50-59, 60-69, 70-79, and 80 years old. BMI was obtained either directly from the recorded BMI values or calculated from weight measurements within the past 10 years and restricted to those taken when the patient was over 16 years old. Obesity was determined according to BMI value, using cut-offs from the US Centers for Disease Control and Prevention: <30 kg/m^2^, not obese; ≥30 and <35 kg/m^2^, class I obesity; ≥35 and <40 kg/m^2^, class II obesity; ≥40 kg/m^2^, class III obesity [9]. Smoking status was grouped into never, former, and current smokers. The Optum data set had race and ethnicity recorded separately. Race included African American, Asian, Caucasian, and other/unknown. Ethnicity included Hispanic, non-Hispanic, or unknown. Due to the fact that Hispanic African American and Hispanic Asian together accounted for only 0.3% of the study population, we treated them simply as African American and Asian, respectively. Caucasian was divided into Hispanic or non-Hispanic White. For Caucasian with unknown ethnicity, we categorized them as unknown race/ethnicity. Consequently, the race/ethnicity variable in the data set was grouped into non-Hispanic White, Hispanic White, African American, and Asian. The regions included West, South, Northeast, and Midwest according to the US Census Bureau.

Based on glycated hemoglobin (HbA_1C_) measured within the past 15 months, diabetes was grouped into uncontrolled (HbA_1C_ ≥58 mmol/mol), controlled (HbA_1C_ <58 mmol/mol), or without recent HbA_1C_ records. Cancers were grouped based on the first diagnosis date (<1 year, ≥1 year). The most recent creatinine value was used to calculate estimated glomerular filtration rate (eGFR) according to the CKD-EPI equation [10]. Since this equation adjusts for race, eGFR was not calculated for patients without race and was considered missing. Reduced kidney function was grouped into eGFR <30 or 30 ≤ eGFR < 60 mL/min/1.73 m^2^. Chronic respiratory disease other than asthma included chronic obstructive pulmonary disease, bronchiectasis, cystic fibrosis, and interstitial lung fibrosis. Cardiac disease included ischemic heart disease and congestive heart failure. Hypertension or high blood pressure was defined as either a prior diagnosis of hypertension or most recent systolic or diastolic blood pressure ≥140 mm Hg or ≥90 mm Hg, respectively. Chronic liver disease included chronic viral hepatitis, cirrhosis, and primary genetic liver disease. Stroke or dementia included hemorrhagic stroke and dementia that were related to cardiovascular etiology. Besides stroke or dementia, other neurological diseases included motor neuron disease, myasthenia gravis, multiple sclerosis, Parkinson disease, cerebral palsy, quadriplegia or hemiplegia, and progressive cerebellar disease. Organ transplant included both solid organ and bone marrow transplant. Autoimmune disease indicated rheumatoid arthritis, systemic lupus erythematosus, and psoriasis. Other immunosuppressive conditions included HIV, permanent immunodeficiency ever diagnosed, as well as aplastic anemia and temporary immunodeficiency diagnosed within the last year.

Information on patients’ comorbidities was obtained by the diagnosis codes in their health care records. The coding system in the Optum COVID-19 data set included ICD-9, ICD-10, and SNOMED CT. To best recapitulate the disease groups as in the OpenSAFELY study, we used the SNOMED code lists provided on the OpenSAFELY website [11] and mapped them to ICD-9/ICD-10 codes using mapping tools from the Unified Medical Language System [12]. The Clinical Classifications Software from the Agency for Healthcare Research and Quality was also used to obtain ICD-9/ICD-10 codes for disease groups where available [13]. All SNOMED CT and ICD-9/ICD-10 codes were compared to the final code lists released by the UK OpenSAFELY platform and were manually curated to match our disease definitions. Decisions on every code list were documented and were reviewed by physicians.

### Statistical Analysis

All statistical analyses mirrored the UK OpenSAFELY study [8], and most of their Stata codes were reused with minor modifications to suit our data set. The Kaplan-Meier method was used to estimate the cumulative incidence of COVID-19–related deaths by age groups and sex. A univariable Cox proportional hazards model was fit for each potential risk factor and was adjusted for age and sex (age-sex adjusted models), with age modeled using a restricted cubic spline. A separate sex-adjusted univariable Cox model for age groups was fitted to show the HRs for different age categories. All the factors including age, sex, obesity, smoking status, region, diabetes, hematological malignancy, lung cancer, other cancers, reduced kidney function, asthma, respiratory disease, chronic cardiac disease, hypertension, liver disease, stroke or dementia, other neurological diseases, organ transplant, rheumatoid arthritis/lupus/psoriasis, and other immunosuppressive conditions were then fit in 1 multivariable Cox proportional hazards model (fully adjusted model). Similarly, age was fit using a restricted cubic spline, and a separate fully adjusted model for age groups was refitted. The proportional hazards assumption was explored by testing for the non-zero slopes of the scaled Schoenfeld residuals for each factor. The Breslow method was used to handle ties, and all the time scales used in the survival analysis were measured in days. Estimated HRs and their 95% CIs are reported for both age-sex adjusted models, as well as the fully adjusted model.

In the primary analysis, participants with missing BMI, smoking status, and eGFR were considered to be non-obese, be never-smokers, and have normal kidney function based on the assumption that having these characteristics were more likely to be captured. A sensitivity analysis was conducted using participants with complete records for these factors only. The differences in HRs were compared with the primary analysis. Due to around 18% of participants without a recorded race, it was not included in the primary model, and its HR was separately obtained by fitting a Cox model using observations with known race. This race-adjusted model, together with other covariates, was presented in another sensitivity analysis to assess the impact of including race on all other factors.

C-statistics were calculated to show the model’s discriminative performance. Due to the computational limits, this was done by randomly sampling 2000 observations from both with and without the event of interest. The process was repeated 10 times, and the average was taken as the estimate of the C-statistic. Weights were applied to the calculation [14]. All *P* values shown here are 2-sided.

Data management was performed in SQL, Python 3.6.10, and R 3.6. All statistical analyses were conducted using Stata/IC 16.1.

## Results

**Figure 1:**
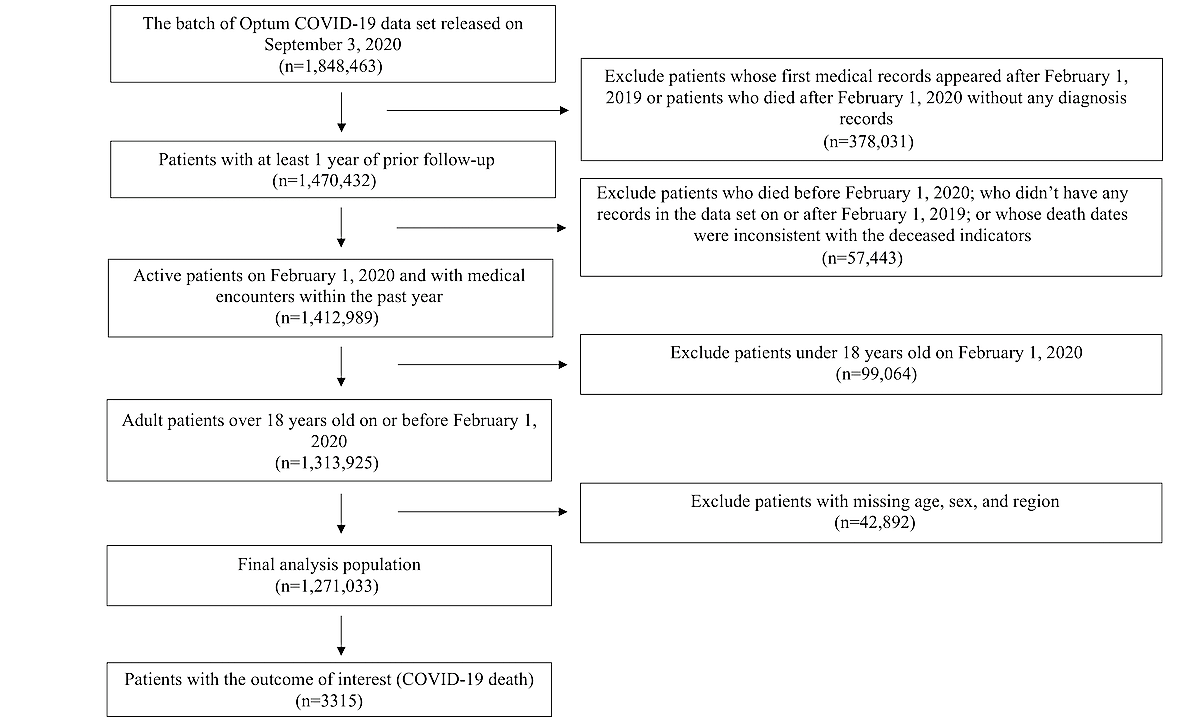
Flowchart for defining the study population in the United States between February 1, 2020, and August 31, 2020.

Among 1,848,463 individuals in the original sample, exclusions were made for the following reasons: lack of a complete year of information prior to February 2020 (378,031/1,848,463, 20.45%), inconsistent or missing information on death (57,443/1,848,463, 3.11%), age less than 18 years (99,064/1,848,463, 5.36%), and missing information on age, sex, or geographic region (42,892/1,848,463, 2.32%), leaving an analytic sample of 1,271,033 persons who were tested for COVID-19 between February 1, 2020, and August 31, 2020 (Figure 1). Among them, 3315 deaths were attributed to COVID-19 by the end of the study. A summary of patient characteristics is shown in Table 1. Certain characteristics had missing information for a proportion of the 1,271,033 persons: BMI, 100,237 (7.89%); smoking status, 88,006 (6.92%); race, 225,881 (17.77%); blood pressure, 79,142 (6.23%); and creatinine, 326,787 (25.71%).

**Figure 2:**
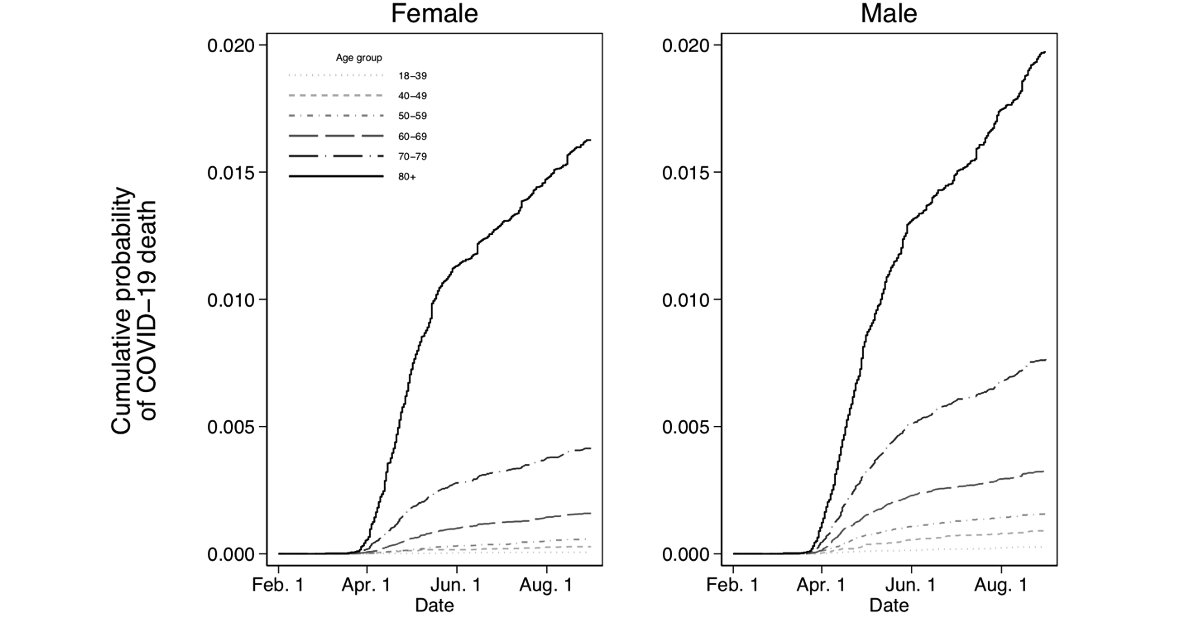
Kaplan-Meier cumulative probability of death due to COVID-19 for (A) women and (B) men.

Kaplan-Meier curves showed that men had a higher cumulative probability of death from COVID-19 in every age group compared with women. In addition, mortality rose as age increased for both sexes (Figure 2).

**Figure 3:**
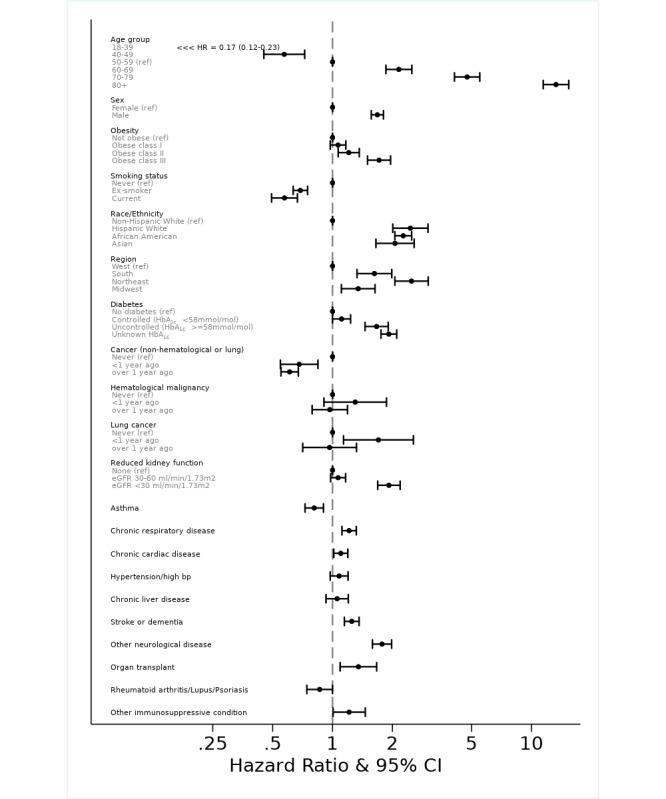
Forest plot showing hazard ratios for each risk factor from the fully adjusted Cox proportional hazards model (n=1,271,033). The values for race were separately ascertained by fitting a Cox proportional hazards model using only those with known race (n=1,045,152) and adjusted for all other covariates. eGFR: estimated glomerular filtration rate.

The HR of COVID-19 deaths for each characteristic is shown in Figure 3 and Table 2.

**Figure 4:**
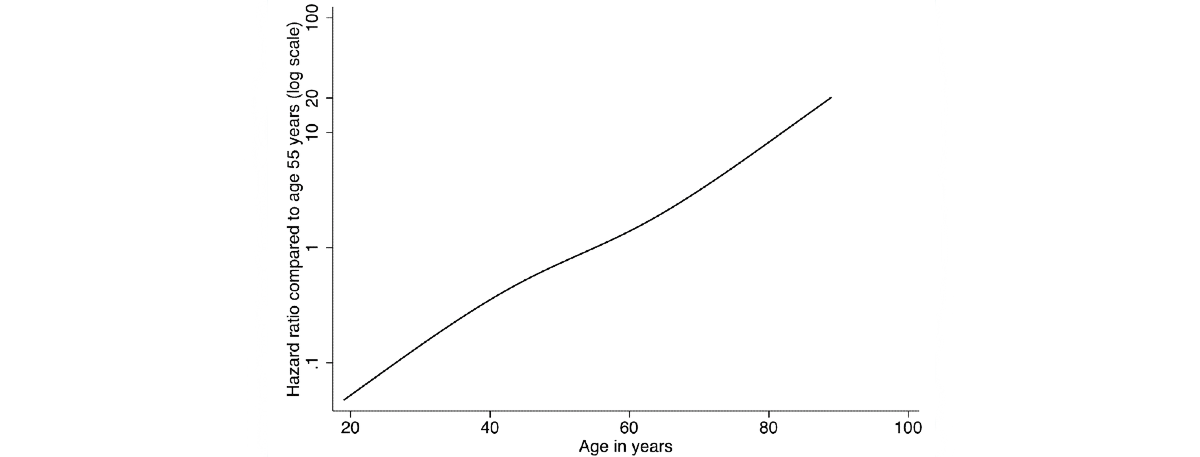
Log-linear relationship between log hazard ratios and age, which was fitted using a restricted cubic spline with 4 knots. The plot was obtained from the fully adjusted model (excluding race) by setting all covariates other than age to the respective reference levels.

In this study, except when reporting the HRs for age groups (fit as a categorical variable), age was otherwise modeled as a restricted cubic spline. With that approach, the relationship between log HRs and age was approximately log-linear (Figure 4). The risks of COVID-19–related death increased in older age groups. In people over 80 years old, the risk was around 13 times that in those aged 50-59 years (reference group). Our results also showed that men had a higher risk of COVID-19–related death compared with women. The minority groups (Hispanic White, African American, and Asian) also had elevated risks compared with non-Hispanic Whites, with HRs ranging from 2.06 (Asians) to 2.46 (Hispanic Whites) in the fully adjusted model. People who lived in the Northeast had the highest HR of 2.50 (2.06-3.03).

The risk increased with rising BMI. Patients with diabetes showed elevated risks. However, well-controlled diabetes (HbA_1c_ <58 mmol/mol during the past 15 months) diminished the risk to 1.11 (1.00-1.23) compared with that of 1.67 (1.46-1.91) in patients with poorer diabetic control or 1.92 (1.75-2.10) in those without recent assessment of control. Other chronic conditions including cardiac disease, severely reduced kidney function (eGFR <30 mL/min/1.73 m^2^), chronic respiratory disease, stroke or dementia, other neurological diseases, organ transplant, and other immunosuppressive conditions were associated with elevated risks of COVID-19–related death. The effects of hematological and lung cancers were investigated separately from other cancers due to their direct impact on the immune system and the sites of the COVID-19 infection, respectively. Other cancers did not increase the risk, whereas having lung cancer diagnosed within the prior year raised the risk of COVID-19–related death. Patients diagnosed with hematological cancer within the prior year had a higher but statistically nonsignificant risk elevation.

Neither former nor current smokers had elevated risks of COVID-19–related death. Instead, the risks were significantly lower compared with nonsmokers. Participants with hypertension showed higher risk in the age-sex adjusted univariable model. However, the HR lost statistical significance when other covariates were included. To investigate which factors contributed to this reduction, we included other variables one at a time to the model containing age, sex, and hypertension. We found that obesity, diabetes, and cardiac disease were primarily responsible for the diminished association. Including these 3 factors in the age-sex adjusted hypertension model reduced the HR for hypertension from 1.30 to 1.03 (0.93-1.14). Similarly, the apparent impact of chronic liver disease was decreased with the adjustment for diabetes. Asthma and autoimmune diseases did not show increased risks of COVID-19–related death in either the age-sex adjusted model or the fully adjusted model.

**Table 1:** Characteristics of the overall adult study population and COVID-19 fatalities in the United States between February 1, 2020, and August 31, 2020.

Characteristic			Overall sample (N=1,271,033), n (%)		Number of COVID-19–related deaths (n=3315), n (%)
**Age (years)**					
	18-39	348,372 (27.41)		46 (0.01)	
	40-49	188,305 (14.82)		96 (0.05)	
	50-59	248,913 (19.58)		246 (0.10)	
	60-69	248,017 (19.51)		578 (0.23)	
	70-79	151,866 (11.95)		869 (0.57)	
	≥80	85,560 (6.73)		1480 (1.73)	
**Sex**					
	Female	763,459 (60.07)		1472 (0.19)	
	Male	507,574 (39.93)		1843 (0.36)	
**BMI (kg/m^2^)**					
	<18.5	16,190 (1.27)		62 (0.38)	
	18.5-24.9	283,597 (22.31)		682 (0.24)	
	25-29.9	356,721 (28.07)		910 (0.26)	
	30-34.9 (obesity class I)	257,837 (20.29)		658 (0.26)	
	35-39.9 (obesity class II)	138,765 (10.92)		320 (0.23)	
	≥40 (obesity class III)	117,686 (9.26)		278 (0.24)	
	Missing	100,237 (7.89)		405 (0.40)	
**Smoking**					
	Never	262,320 (20.64)		574 (0.22)	
	Former	727,211 (57.21)		2163 (0.30)	
	Current	193,496 (15.22)		236 (0.12)	
	Missing	88,006 (6.92)		342 (0.39)	
**Race/ethnicity**					
	Non-Hispanic White	837,195 (65.87)		1933 (0.23)	
	Hispanic White	30,582 (2.41)		99 (0.32)	
	African American	147,830 (11.63)		564 (0.38)	
	Asian	29,545 (2.32)		85 (0.29)	
	Missing	225,881 (17.77)		634 (0.28)	
**Region**					
	West	100,986 (7.95)		112 (0.11)	
	South	225,884 (17.77)		664 (0.29)	
	Northeast	389,344 (30.63)		1449 (0.37)	
	Midwest	554,819 (43.65)		1090 (0.20)	
**Blood pressure**					
	Normal^a^	373,078 (29.35)		661 (0.18)	
	Elevated^b^	184,987 (14.55)		487 (0.26)	
	High, stage I^c^	467,196 (36.76)		1064 (0.23)	
	High, stage II^d^	166,630 (13.11)		788 (0.47)	
	Missing	79,142 (6.23)		315 (0.40)	
**High blood pressure/hypertension**					
	Yes	650,425 (51.17)		2738 (0.42)	
	No	620,608 (48.83)		577 (0.09)	
**Chronic respiratory disease**					
	Yes	170,033 (13.38)		1017 (0.60)	
	No	1,101,000 (86.62)		2298 (0.21)	
**Asthma**					
	Yes	208,254 (16.38)		422 (0.20)	
	No	1,062,779 (83.62)		2893 (0.27)	
**Cardiac disease**					
	Yes	215,816 (16.98)		1583 (0.73)	
	No	1,055,217 (83.02)		1732 (0.16)	
**Diabetes**					
	HbA_1c_^e^ <58 mmol/mol	105,697 (8.32)		497 (0.47)	
	HbA_1c_ ≥58 mmol/mol	49,193 (3.87)		267 (0.54)	
	No recent^f^ HbA_1c_ value	86,896 (6.84)		699 (0.80)	
	Not diabetic	1,029,247 (80.98)		1852 (0.18)	
**Other cancer (excluding hematological and lung cancer)**					
	Diagnosed <1 year	30,835 (2.43)		85 (0.28)	
	Diagnosed ≥1 year	137,456 (10.81)		467 (0.34)	
	Never	1,102,742 (86.76)		2763 (0.25)	
**Hematological cancer**					
	Diagnosed <1 year	4681 (0.37)		30 (0.64)	
	Diagnosed ≥1 year	17,873 (1.41)		98 (0.55)	
	Never	1,248,479 (98.23)		3187 (0.26)	
**Lung cancer**					
	Diagnosed <1 year	2927 (0.23)		24 (0.82)	
	Diagnosed ≥1 year	7419 (0.58)		41 (0.55)	
	Never	1,260,687 (99.19)		3250 (0.26)	
**eGFR^g^ (mL/min/1.73 m^2^)^h^**					
	≥60	823,048 (64.75)		1378 (0.17)	
	45-59.9	71,698 (5.64)		499 (0.70)	
	30-44.9	30,453 (2.40)		361 (1.19)	
	15-29.9	11,007 (0.87)		172 (1.56)	
	<15	8040 (0.63)		119 (1.48)	
	Missing	326,787 (25.71)		786 (0.24)	
**Chronic liver disease**					
	Yes	90,213 (7.10)		265 (0.29)	
	No	1,180,820 (92.90)		3050 (0.26)	
**Stroke or dementia**					
	Yes	104,876 (8.25)		913 (0.87)	
	No	1,166,157 (91.75)		2402 (0.21)	
**Other neurological diseases**					
	Yes	41,187 (3.24)		391 (0.95)	
	No	1,229,846 (96.76)		2924 (0.24)	
**Organ transplant**					
	Yes	12,429 (0.98)		95 (0.76)	
	No	1,258,604 (99.02)		3220 (0.26)	
**RA^i^, SLE^j^, or psoriasis**					
	Yes	65,387 (5.14)		188 (0.29)	
	No	1,205,646 (94.86)		3127 (0.26)	
**Other immunosuppressive condition**					
	Yes	31,005 (2.44)		127 (0.41)	
	No	1,240,028 (97.56)		3188 (0.26)	

^a^Systolic blood pressure <120 mm Hg; diastolic blood pressure <80 mm Hg.

^b^Systolic blood pressure ≥120 and ≤129 mm Hg; diastolic blood pressure <80.

^c^Systolic blood pressure ≥130 and ≤139 mm Hg; diastolic blood pressure ≥80 and ≤89 mm Hg.

^d^Systolic blood pressure ≥140 or diastolic blood pressure ≥90.

^e^HbA_1c_: glycated hemoglobin.

^f^HbA_1c_ value within 15 months before February 1, 2020.

^g^eGFR: estimated glomerular filtration rate.

^h^Calculated from the creatinine value.

^i^RA: rheumatoid arthritis.

^j^SLE: systemic lupus erythematosus.

**Table 2:** Adjusted hazard ratios (HRs) for COVID-19–related death for each patient characteristic.

Characteristic		Age-sex adjusted model^a^, HR (95% CI)	Fully adjusted model (primary analysis)^b^, HR (95% CI)
**Age^c^ (years)**			
	18-39	0.14 (0.10-0.19)	0.17 (0.12-0.23)
	40-49	0.53 (0.42-0.67)	0.57 (0.45-0.72)
	50-59	1.00 (ref^d^)	1.00 (ref)
	60-69	2.32 (2.00-2.69)	2.15 (1.85-2.50)
	70-79	5.70 (4.95-6.57)	4.75 (4.10-5.50)
	≥80	18.00 (15.73-20.60)	13.28 (11.46-15.39)
**Sex**			
	Female	1.00 (ref)	1.00 (ref)
	Male	1.68 (1.57-1.80)	1.68 (1.57-1.80)
**Obesity**			
	Not obese	1.00 (ref)	1.00 (ref)
	Class I (BMI 30-34.9 kg/m^2^)	1.07 (0.98-1.17)	1.07 (0.97-1.17)
	Class II (BMI 35-39.9 kg/m^2^)	1.25 (1.11-1.41)	1.21 (1.07-1.36)
	Class III (BMI ≥40 kg/m^2^)	1.83 (1.61-2.09)	1.71 (1.50-1.96)
**Smoking**			
	Never	1.00 (ref)	1.00 (ref)
	Former	0.76 (0.70-0.82)	0.69 (0.63-0.75)
	Current	0.64 (0.55-0.73)	0.57 (0.49-0.67)
**Race/ethnicity**			
	Non-Hispanic White	1.00 (ref)	1.00 (ref)
	Hispanic White	2.76 (2.25-3.38)	2.46 (2.01-3.02)
	African American	2.65 (2.41-2.91)	2.27 (2.06-2.50)
	Asian	2.30 (1.85-2.85)	2.06 (1.65-2.57)
**Region**			
	West	1.00 (ref)	1.00 (ref)
	South	1.85 (1.52-2.27)	1.62 (1.33-1.98)
	Northeast	2.63 (2.17-3.18)	2.50 (2.06-3.03)
	Midwest	1.49 (1.23-1.81)	1.35 (1.11-1.64)
High blood pressure/hypertension		1.30 (1.18-1.42)	1.08 (0.97-1.20)
Chronic respiratory disease		1.25 (1.16-1.35)	1.21 (1.12-1.32)
Asthma		0.90 (0.81-1.00)	0.81 (0.73-0.90)
Cardiac disease		1.34 (1.24-1.44)	1.10 (1.01-1.19)
**Diabetes**			
	HbA_1c_^e^ <58 mmol/mol	1.28 (1.16-1.41)	1.11 (1.00-1.23)
	HbA_1c_ ≥58 mmol/mol	1.99 (1.75-2.26)	1.67 (1.46-1.91)
	No recent^f^ HbA_1c_ value	2.19 (2.01-2.39)	1.92 (1.75-2.10)
**Other cancer (excluding hematological and lung cancer)**			
	Diagnosed <1 year	0.71 (0.58-0.89)	0.68 (0.55-0.84)
	Diagnosed ≥1 year	0.65 (0.59-0.72)	0.61 (0.55-0.67)
**Hematological cancer**			
	Diagnosed <1 year	1.36 (0.95-1.94)	1.30 (0.91-1.87)
	Diagnosed ≥1 year	1.02 (0.83-1.25)	0.97 (0.79-1.19)
**Lung cancer**			
	Diagnosed <1 year	1.61 (1.08-2.40)	1.70 (1.14-2.55)
	Diagnosed ≥1 year	0.92 (0.68-1.25)	0.97 (0.71-1.32)
**Reduced kidney function^g^**			
	eGFR^h^ 30-60 mL/min^1^/1.73 m^2^	1.12 (1.03-1.22)	1.07 (0.98-1.16)
	eGFR <30 mL/min^1^/1.73 m^2^	2.35 (2.07-2.66)	1.92 (1.69-2.19)
Chronic liver disease		1.19 (1.05-1.35)	1.05 (0.93-1.20)
Stroke or dementia		1.44 (1.33-1.56)	1.25 (1.15-1.36)
Other neurological diseases		1.92 (1.72-2.13)	1.77 (1.59-1.98)
Organ transplant		1.66 (1.35-2.03)	1.35 (1.09-1.67)
RA^i^, SLE^j^, or psoriasis		0.89 (0.77-1.03)	0.86 (0.74-1.00)
Other immunosuppressive condition		1.35 (1.13-1.62)	1.21 (1.01-1.46)

^a^Univariable Cox proportional hazards model adjusted for age and sex.

^b^Multivariable Cox proportional hazards model containing all covariates other than race; hazard ratios for race were obtained from a separate model using only observations with known race. Missing BMI, smoking status, and estimated glomerular filtration rate (eGFR) were considered to be nonobese, never smokers, and with normal kidney function.

^c^For all models, age was modeled as a restricted cubic spline except for age groups.

^d^ref: reference level.

^e^HBA_1c_: glycated hemoglobin.

^f^HbA_1c_ values within 15 months before February 1, 2020.

^g^Calculated from the creatinine value.

^h^eGFR: estimated glomerular filtration rate

^i^RA: rheumatoid arthritis.

^j^SLE: systemic lupus erythematosus.

**Figure 5:**
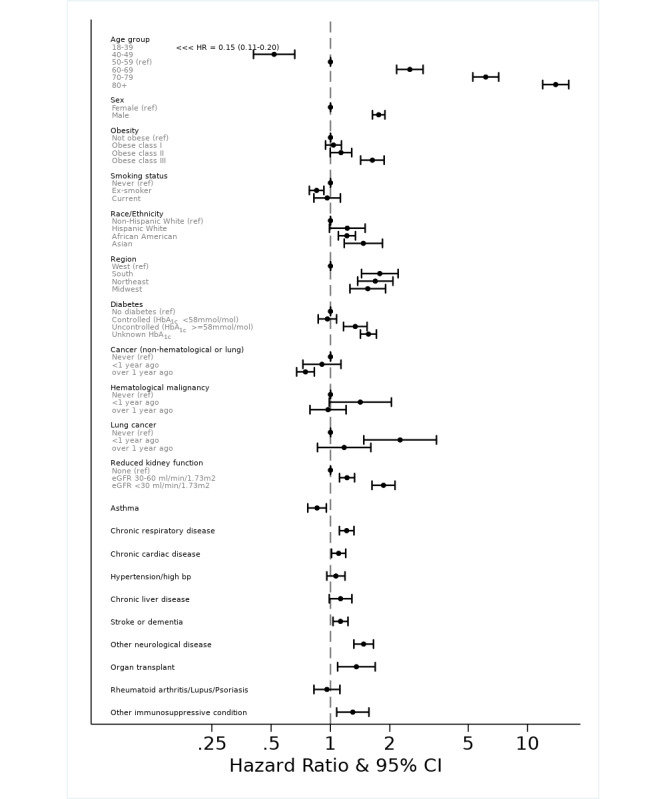
Forest plot showing the hazard ratios (HRs) for each risk factor from the fully adjusted Cox proportional hazards model among COVID-19 patients (n=116,426). The values for race were separately ascertained by fitting a Cox proportional hazards model using only those with known race (n=89,027) and adjusted for all other covariates. The number of COVID-19–related deaths is slightly different from the full cohort (3136 among COVID-19 patients vs 3315 among the full cohort) due to failing or censoring on the same day as being diagnosed with COVID-19. eGFR: estimated glomerular filtration rate.

In the primary analysis, all the eligible participants were included regardless of SARS-CoV-2 test results. To investigate if the risk factors for death among persons with COVID-19 differed from mortality in the overall cohort, separate Cox proportional hazard models using only the lab-confirmed COVID-19 cases were fit (Figure 5 and Tables 3 and 4). Most of the findings did not differ greatly from the full cohort analysis. The lower risks among current smokers and persons with other cancers diagnosed within 1 year seen in the primary analysis, however, were eliminated. In addition, the magnitudes of the risk elevations for minority groups were reduced among the COVID-19 patients.

**Table 3:** Characteristics of the subpopulation of COVID-19–confirmed cases.

Characteristic			Overall sample (n=116,426), n (%)		Number of COVID-19–related deaths (n=3136^a^), n (%)
**Age (years)**					
	18-39	35,207 (30.24)		43 (0.12)	
	40-49	19,344 (16.61)		93 (0.48)	
	50-59	22,968 (19.73)		233 (1.01)	
	60-69	19,409 (16.67)		548 (2.82)	
	70-79	10,949 (9.40)		827 (7.55)	
	≥80	8549 (7.34)		1392 (16.28)	
**Sex**					
	Female	67,953 (58.37)		1388 (2.04)	
	Male	48,473 (41.63)		1748 (3.61)	
**BMI (kg/m^2^)**					
	<18.5	1142 (0.98)		58 (5.08)	
	18.5-24.9	22,891 (19.66)		650 (2.84)	
	25-29.9	31,680 (27.21)		869 (2.74)	
	30-34.9 (obesity class I)	24,232 (20.81)		621 (2.56)	
	35-39.9 (obesity class II)	13,543 (11.63)		303 (2.24)	
	≥40 (obesity class III)	11,752 (10.09)		266 (2.26)	
	Missing	11,186 (9.61)		369 (3.30)	
**Smoking**					
	Never	29,452 (25.30)		553 (1.88)	
	Former	66,351 (56.99)		2053 (3.09)	
	Current	10,917 (9.38)		218 (2.00)	
	Missing	9706 (8.34)		312 (3.21)	
**Race/ethnicity**					
	Non-Hispanic White	59,162 (50.82)		1833 (3.10)	
	Hispanic White	5351 (4.60)		96 (1.79)	
	African American	21,176 (18.19)		536 (2.53)	
	Asian	3338 (2.87)		83 (2.49)	
	Missing	27,399 (23.53)		588 (2.15)	
**Region**					
	West	9941 (8.54)		98 (0.99)	
	South	19,490 (16.74)		633 (3.25)	
	Northeast	41,808 (35.91)		1381 (3.30)	
	Midwest	45,187 (38.81)		1024 (2.27)	
**Blood pressure**					
	Normal^b^	33,932 (29.14)		628 (1.85)	
	Elevated^c^	16,512 (14.18)		463 (2.80)	
	High, stage I^d^	42,219 (36.26)		1024 (2.43)	
	High, stage II^e^	15,406 (13.23)		734 (4.76)	
	Missing	8357 (7.18)		287 (3.43)	
**High blood pressure/hypertension**					
	Yes	57,048 (49.00)		2599 (4.56)	
	No	59,378 (51.00)		537 (0.90)	
**Chronic respiratory disease**					
	Yes	13,007 (11.17)		966 (7.43)	
	No	103,419 (88.83)		2170 (2.10)	
**Asthma**					
	Yes	17,140 (14.72)		407 (2.37)	
	No	99,286 (85.28)		2729 (2.75)	
**Cardiac disease**					
	Yes	18,035 (15.49)		1497 (8.30)	
	No	98,391 (84.51)		1639 (1.67)	
**Diabetes**					
	HbA_1c_^f^ <58 mmol/mol	9822 (8.44)		476 (4.85)	
	HbA_1c_ ≥58 mmol/mol	5207 (4.47)		251 (4.82)	
	No recent^g^ HbA_1c_ value	8879 (7.63)		661 (7.44)	
	Not diabetic	92,518 (79.47)		1748 (1.89)	
**Other cancer (excluding hematological and lung cancer)**					
	Diagnosed <1 year	2294 (1.97)		82 (3.57)	
	Diagnosed ≥1 year	10,791 (9.27)		453 (4.20)	
	Never	103,341 (88.76)		2601 (2.52)	
**Hematological cancer**					
	Diagnosed <1 year	395 (0.34)		30 (7.59)	
	Diagnosed ≥1 year	1433 (1.23)		92 (6.42)	
	Never	114,598 (98.43)		3014 (2.63)	
**Lung cancer**					
	Diagnosed <1 year	170 (0.15)		22 (12.94)	
	Diagnosed ≥1 year	506 (0.43)		41 (8.10)	
	Never	115,750 (99.42)		3073 (2.65)	
**eGFR^h^ (mL/min/1.73 m^2^)^i^**					
	≥60	67,744 (58.29)		1317 (1.94)	
	45-59.9	5574 (4.79)		479 (8.59)	
	30-44.9	2497 (2.14)		340 (13.62)	
	15-29.9	1003 (0.86)		159 (15.85)	
	<15	888 (0.76)		113 (12.73)	
	Missing	38,720 (33.26)		728 (1.88)	
**Chronic liver disease**					
	Yes	7367 (6.33)		256 (3.47)	
	No	109,059 (93.67)		2880 (2.64)	
**Stroke or dementia**					
	Yes	9311 (8.00)		866 (9.30)	
	No	107,115 (92.00)		2270 (2.12)	
**Other neurological diseases**					
	Yes	3887 (3.34)		368 (9.47)	
	No	112,539 (96.66)		2768 (2.46)	
**Organ transplant**					
	Yes	1090 (0.94)		90 (8.26)	
	No	115,336 (99.06)		3046 (2.64)	
**RA^j^, SLE^k^, or psoriasis**					
	Yes	4989 (4.29)		181 (3.63)	
	No	111,437 (95.71)		2955 (2.65)	
**Other immunosuppressive condition**					
	Yes	2528 (2.17)		126 (4.98)	
	No	113,898 (97.83)		3010 (2.64)	

^a^179 deaths were excluded, compared with the full cohort, due to failing or censoring on the same day as being diagnosed with COVID-19.

^b^Systolic blood pressure <120 mm Hg; diastolic blood pressure <80 mm Hg.

^c^Systolic blood pressure ≥120 and ≤129 mm Hg; diastolic blood pressure <80.

^d^Systolic blood pressure ≥130 and ≤139 mm Hg; diastolic blood pressure ≥80 and ≤89 mm Hg.

^e^Systolic blood pressure ≥140 or diastolic blood pressure ≥90.

^f^HbA_1c_: glycated hemoglobin.

^g^HbA_1c_ value within 15 months before February 1, 2020.

^h^eGFR: estimated glomerular filtration rate.

^i^Calculated from the creatinine value.

^j^RA: rheumatoid arthritis.

^k^SLE: systemic lupus erythematosus.

**Table 4:** Adjusted hazard ratios (HRs) for COVID-19–related death among COVID-19–confirmed cases.

Characteristic		Age-sex adjusted model^a^, HR (95% CI)	Fully adjusted model^b^, HR (95% CI)
**Age^c^ (years)**			
	18-39	0.13 (0.09-0.17)	0.15 (0.11-0.20)
	40-49	0.49 (0.38-0.62)	0.52 (0.41-0.66)
	50-59	1.00 (ref^d^)	1.00 (ref)
	60-69	2.76 (2.37-3.22)	2.53 (2.17-2.95)
	70-79	7.62 (6.59-8.81)	6.13 (5.28-7.13)
	≥80	18.51 (16.11-21.26)	13.89 (11.93-16.17)
**Sex**			
	Female	1.00 (ref)	1.00 (ref)
	Male	1.76 (1.64-1.88)	1.76 (1.63-1.89)
**Obesity**			
	Not obese	1.00 (ref)	1.00 (ref)
	Class I (BMI 30-34.9 kg/m^2^)	1.05 (0.96-1.15)	1.04 (0.94-1.14)
	Class II (BMI 35-39.9 kg/m^2^)	1.20 (1.06-1.36)	1.13 (1.00-1.28)
	Class III (BMI ≥40 kg/m^2^)	1.79 (1.57-2.05)	1.63 (1.42-1.87)
**Smoking**			
	Never	1.00 (ref)	1.00 (ref)
	Former	0.98 (0.90-1.06)	0.85 (0.78-0.93)
	Current	1.15 (0.99-1.33)	0.96 (0.82-1.12)
**Race/ethnicity**			
	Non-Hispanic White	1.00 (ref)	1.00 (ref)
	Hispanic White	1.30 (1.06-1.59)	1.22 (0.99-1.50)
	African American	1.31 (1.19-1.44)	1.21 (1.10-1.34)
	Asian	1.35 (1.08-1.69)	1.47 (1.17-1.84)
**Region**			
	West	1.00 (ref)	1.00 (ref)
	South	2.00 (1.62-2.48)	1.78 (1.44-2.20)
	Northeast	1.77 (1.44-2.17)	1.69 (1.37-2.07)
	Midwest	1.68 (1.36-2.07)	1.55 (1.26-1.90)
High blood pressure/hypertension		1.35 (1.22-1.48)	1.07 (0.96-1.18)
Chronic respiratory disease		1.34 (1.24-1.45)	1.21 (1.11-1.32)
Asthma		0.97 (0.87-1.08)	0.86 (0.77-0.95)
Cardiac disease		1.35 (1.25-1.45)	1.10 (1.01-1.19)
**Diabetes**			
	HbA_1c_^e^ <58 mmol/mol	1.16 (1.05-1.28)	0.96 (0.87-1.07)
	HbA_1c_ ≥58 mmol/mol	1.63 (1.43-1.86)	1.34 (1.16-1.53)
	No recent^f^ HbA_1c_ value	1.80 (1.64-1.97)	1.56 (1.42-1.71)
**Other cancer (excluding hematological and lung cancer)**			
	Diagnosed <1 year	0.94 (0.75-1.17)	0.91 (0.72-1.13)
	Diagnosed ≥1 year	0.80 (0.73-0.89)	0.75 (0.67-0.83)
**Hematological cancer**			
	Diagnosed <1 year	1.57 (1.10-2.25)	1.42 (0.99-2.04)
	Diagnosed ≥1 year	1.04 (0.84-1.28)	0.97 (0.79-1.20)
**Lung cancer**			
	Diagnosed <1 year	2.41 (1.58-3.67)	2.26 (1.48-3.45)
	Diagnosed ≥1 year	1.19 (0.87-1.62)	1.17 (0.86-1.60)
**Reduced kidney function^g^**			
	eGFR^h^ 30-60 mL/min/1.73 m^2^	1.29 (1.18-1.41)	1.21 (1.11-1.33)
	eGFR <30 mL/min/1.73 m^2^	2.22 (1.95-2.53)	1.86 (1.62-2.13)
Chronic liver disease		1.28 (1.13-1.46)	1.13 (0.99-1.28)
Stroke or dementia		1.33 (1.23-1.45)	1.12 (1.03-1.23)
Other neurological diseases		1.61 (1.44-1.80)	1.47 (1.31-1.65)
Organ transplant		1.73 (1.40-2.13)	1.35 (1.09-1.69)
RA^i^, SLE^j^, or psoriasis		1.02 (0.88-1.18)	0.96 (0.82-1.12)
Other immunosuppressive condition		1.52 (1.27-1.82)	1.30 (1.08-1.57)

^a^Univariable Cox proportional hazard model adjusted for age and sex.

^b^Multivariable Cox proportional hazards model containing all covariates other than race; hazard ratios for race were obtained from a separate model using only observations with known race. Missing BMI, smoking status, and estimated glomerular filtration rate (eGFR) were considered to be nonobese, never smokers, and with normal kidney function.

^c^For all models, age was modeled as a restricted cubic spline except for age groups.

^d^ref: reference level.

^e^HbA_1c_: glycated hemoglobin.

^f^HbA_1c_ values within 15 months before February 1, 2020.

^g^Calculated from the creatinine value.

^h^eGFR: estimated glomerular filtration rate

^i^RA: rheumatoid arthritis.

^j^SLE: systemic lupus erythematosus.

In the primary analysis, participants with missing BMI, smoking status, and eGFR were treated as non-obese, never-smoker, and normal kidney function. To determine whether this affected the results, we fit a separate Cox proportional hazard model using only the participants with complete information on all 3 factors. The HRs were similar to those from the primary analysis, suggesting robustness of the model to missing values (Table 5).

Similarly, due to about 18% of the participants missing a designation of race, the primary multivariable model did not include race. The HRs reported in the primary analysis (Table 2) for race were obtained by a separate Cox proportional hazard model using complete records of race/ethnicity only. HRs for other factors in this model were very similar to those obtained from the primary analysis, suggesting that including race/ethnicity did not alter the model meaningfully (Table 5). The proportional hazards assumption violation was detected for some variables in the primary model (*P*<.001). Checking the plots of the scaled Schoenfeld residuals versus time, however, revealed no non-zero slopes in any of the factors. The apparent violation of the proportional hazards assumption, therefore, could be due to the large sample size of the study. The C-statistic of the primary model was 0.87, demonstrating a satisfactory discriminative ability in identifying the risks of COVID-19–related death.

**Table 5:** Sensitivity analyses for the Cox proportional hazards model under various conditions.

Characteristic			Primary analysis (n=1,271,033), HR^a,b^ (95% CI)		Cases complete with BMI, smoking status, and eGFR^c^ (n=906,359), HR^b^ (95% CI)		Cases complete with race/ethnicity (n=1,045,152), HR^b^ (95% CI)
Number of outcomes, n			3315		2342		2681
**Age^d^ (years)**							
	18-39	0.17 (0.12-0.23)		0.24 (0.16-0.37)		0.15 (0.10-0.23)	
	40-49	0.57 (0.45-0.72)		0.56 (0.41-0.77)		0.53 (0.40-0.71)	
	50-59	1.00 (ref^e^)		1.00 (ref)		1.00 (ref)	
	60-69	2.15 (1.85-2.50)		1.96 (1.63-2.37)		2.27 (1.91-2.71)	
	70-79	4.75 (4.10-5.50)		4.28 (3.56-5.15)		5.32 (4.48-6.30)	
	≥80	13.28 (11.46-15.39)		12.11 (10.05-14.59)		15.88 (13.37-18.86)	
**Sex**							
	Female	1.00 (ref)		1.00 (ref)		1.00 (ref)	
	Male	1.68 (1.57-1.80)		1.56 (1.43-1.69)		1.73 (1.60-1.87)	
**Obesity**							
	Not obese	1.00 (ref)		1.00 (ref)		1.00 (ref)	
	Class I (BMI 30-34.9 kg/m^2^)	1.07 (0.97-1.17)		1.08 (0.97-1.20)		1.02 (0.92-1.13)	
	Class II (BMI 35-39.9 kg/m^2^)	1.21 (1.07-1.36)		1.20 (1.04-1.38)		1.23 (1.07-1.41)	
	Class III (BMI ≥40 kg/m^2^)	1.71 (1.50-1.96)		1.68 (1.44-1.96)		1.75 (1.51-2.03)	
**Smoking**							
	Never	1.00 (ref)		1.00 (ref)		1.00 (ref)	
	Former	0.69 (0.63-0.75)		0.85 (0.75-0.95)		0.75 (0.68-0.82)	
	Current	0.57 (0.49-0.67)		0.72 (0.60-0.86)		0.62 (0.53-0.74)	
**Race/ethnicity**							
	Non-Hispanic White	1.00 (ref)		1.00 (ref)		1.00 (ref)	
	Hispanic White	2.46 (2.01-3.02)		2.72 (2.19-3.37)		2.46 (2.01-3.02)	
	African American	2.27 (2.06-2.50)		2.20 (1.97-2.45)		2.27 (2.06-2.50)	
	Asian	2.06 (1.65-2.57)		2.04 (1.58-2.64)		2.06 (1.65-2.57)	
**Region**							
	West	1.00 (ref)		1.00 (ref)		1.00 (ref)	
	South	1.62 (1.33-1.98)		1.95 (1.49-2.56)		1.51 (1.14-2.01)	
	Northeast	2.50 (2.06-3.03)		2.92 (2.24-3.80)		2.26 (1.71-3.00)	
	Midwest	1.35 (1.11-1.64)		1.64 (1.26-2.13)		1.30 (0.98-1.72)	
High blood pressure/hypertension			1.08 (0.97-1.20)		1.31 (1.13-1.53)		1.05 (0.93-1.18)
Chronic respiratory disease			1.21 (1.12-1.32)		1.23 (1.12-1.35)		1.23 (1.12-1.35)
Asthma			0.81 (0.73-0.90)		0.82 (0.73-0.92)		0.81 (0.72-0.90)
Cardiac disease			1.10 (1.01-1.19)		1.20 (1.09-1.32)		1.14 (1.04-1.25)
**Diabetes**							
	HbA_1c_^f^ <58 mmol/mol	1.11 (1.00-1.23)		1.11 (0.99-1.24)		0.98 (0.88-1.11)	
	HbA_1c_ ≥58 mmol/mol	1.67 (1.46-1.91)		1.63 (1.40-1.89)		1.44 (1.24-1.67)	
	No recent^g^ HbA_1c_ value	1.92 (1.75-2.10)		1.85 (1.66-2.07)		1.64 (1.48-1.81)	
**Other cancer (excluding hematological and lung cancer)**							
	Diagnosed <1 year	0.68 (0.55-0.84)		0.72 (0.57-0.92)		0.73 (0.57-0.92)	
	Diagnosed ≥1 year	0.61 (0.55-0.67)		0.63 (0.56-0.70)		0.62 (0.56-0.69)	
**Hematological cancer**							
	Diagnosed <1 year	1.30 (0.91-1.87)		1.45 (0.99-2.12)		1.32 (0.89-1.96)	
	Diagnosed ≥1 year	0.97 (0.79-1.19)		1.07 (0.86-1.32)		1.06 (0.86-1.31)	
**Lung cancer**							
	Diagnosed <1 year	1.70 (1.14-2.55)		1.91 (1.26-2.88)		1.63 (1.05-2.54)	
	Diagnosed ≥1 year	0.97 (0.71-1.32)		0.91 (0.64-1.28)		1.05 (0.76-1.45)	
**Reduced kidney function^h^**							
	eGFR 30-60 mL/min/1.73 m^2^	1.07 (0.98-1.16)		1.21 (1.10-1.33)		1.17 (1.07-1.28)	
	eGFR 30 mL/min/1.73 m^2^	1.92 (1.69-2.19)		2.15 (1.87-2.47)		1.93 (1.68-2.21)	
Chronic liver disease			1.05 (0.93-1.20)		1.14 (0.99-1.31)		1.11 (0.96-1.27)
Stroke/ dementia			1.25 (1.15-1.36)		1.32 (1.20-1.45)		1.29 (1.18-1.41)
Other neurological diseases			1.77 (1.59-1.98)		1.75 (1.55-1.98)		1.82 (1.61-2.05)
Organ transplant			1.35 (1.09-1.67)		1.25 (1.00-1.58)		1.25 (0.99-1.58)
RA^i^, SLE^j^, or psoriasis			0.86 (0.74-1.00)		0.89 (0.76-1.05)		0.87 (0.74-1.03)
Other immunosuppressive condition			1.21 (1.01-1.46)		1.17 (0.96-1.44)		1.13 (0.92-1.38)

^a^HR: hazard ratio.

^b^Fully adjusted HR.

^c^eGFR: estimated glomerular filtration rate.

^d^For all models, age was modeled as a restricted cubic spline except for age groups.

^e^ref: reference level.

^f^HbA_1c_: glycated hemoglobin.

^g^HbA_1c_ values within 15 months before February 1, 2020.

^h^Calculated from the creatinine value.

^i^RA: rheumatoid arthritis.

^j^SLE: systemic lupus erythematosus.

## Discussion

### Principal Findings

This study, using individual-level EHR data from a large population, is one of the largest cohort studies published on this topic in the United States. The results are complementary to those reported from the N3C—the largest US-based cohort to date.

The inclusion of all populations in the primary analysis may raise the doubt of assessing risks for being infected and for death after infection. Our analysis among COVID-19 patients eliminates the concern by showing no apparent differences between the 2 groups.

Our results regarding increasing risks for demographic factors and comorbidities were consistent with various studies [2,6,8,15,16]. In either the full cohort or the COVID-19–positive cases, the risks for the minority groups only reduced slightly in the fully adjusted models compared with the age-sex adjusted models. This was similar to the OpenSAFELY study, in which including other covariates only explained a small portion of the risks expressed by race/ethnicity. This suggested that other socioeconomic factors, such as income, education, housing, and occupation, could play critical roles.

The elevated risk observed in the Northeast was likely due to the fact that, at the earliest stage of the pandemic, it was the disease hot zone where hospital capacity was stretched and evidence-based disease management protocols were not yet developed. Studies that have investigated the relationship between smoking and COVID-19 prevalence or mortality have generated conflicting results. Some suggested that smoking was not associated with COVID-19 [17,18], while others reported that smoking could lead up to twice the risk of COVID-19–related death [19]. Our results from the full or COVID-19–positive cohorts did not support any increased risks associated with smoking. Lower risks of current and prior smokers were observed in the full cohort, but this might be attributable to smokers perceiving that their risks were higher and thus moderating their risk of viral exposure, rather than to any effect of smoking per se. Studies designed to specially assess the impact of smoking are required to draw a more definitive conclusion.

Many studies have reported a higher risk of severe outcomes of COVID-19 in cancer patients [5,8,20-22]. When examined by cancer types, however, studies in Italy and China showed that lung cancer and hematological malignancies had statistically higher risks of COVID-19–related death, while others did not [20,23]. Our results for cancer were comparable to these findings in which lung cancers and hematological malignancies showed elevated risks, although the HR for hematological malignancies was not statistically significant. Asthma was not associated with an increased risk of COVID-19–related death in this study, which confirms other reports [24,25]. We attempted to stratify asthma based on recent use of oral corticosteroids (OCS) as a proxy for severity, but the limited number of participants (n=333 for recent use of OCS) did not allow a precise estimate.

The prevalence of the comorbidities examined in our study was slightly higher than the National Health Interview Survey 2017 data, as shown in a recent study on a similar topic [26]. This was expected since the present sample was drawn from persons who perceived their risk of COVID-19 to be sufficiently high to warrant testing. A prior report from the Optum COVID-19 data set, although based upon differences in sampling dates and inclusion criteria, yielded similar prevalence estimates to this analysis [27].

### Limitations

The current study has some important limitations that must be considered. Unlike the UK’s National Health Service, the fragmented health care system in the United States limits the ability to aggregate health records for the general US population. Patients switching to different insurance companies or providers may experience gaps in their electronic medical records, and those without health insurance are much less likely to be seen by the providers contributing to this cohort. The geographic distribution of the participating providers also can affect the representativeness of this sample.

To correctly capture patients’ baseline health conditions, we imposed the restriction that only participants with at least 1 year of prior health care engagement were eligible to be included in this study. However, this approach may introduce some selection biases. Those who did not meet this criterion may have different outcomes from COVID-19 infection and therefore, could reduce the generalizability of our study findings.

To protect the identities of individual patients, the exact death date and the cause of death were not included in the data set. Although we have developed a reasonable criterion to define the 2 features (see the Methods section), misestimations are still possible, impacting the risk assessments. In addition, at the early stage of the pandemic, testing and diagnosis coding standards were not fully established, so some persons with COVID-19 may not have been detected. Such misclassification, to the extent that it occurred, would tend to be random and only serve to reduce the associations that were observed.

### Conclusion

Identifying patient characteristics associated with increased risks of COVID-19–related death has been an important topic since the start of the pandemic. Using over 1 million patient EHRs to conduct a large cohort survival analysis, we found that age, gender, race, region, and comorbidities including obesity, diabetes, recently diagnosed lung cancer, reduced kidney function, chronic respiratory disease, cardiac disease, stroke or dementia, other neurological diseases, organ transplant, and other immunosuppressive conditions were associated with elevated risks of COVID-19–related death, while smoking, other cancers, asthma, and certain autoimmune diseases were not. Our large and geographically diversified individual-level data provide comprehensive and reliable results on this topic. This study can also serve as a foundation for future policy making about the protection of vulnerable populations, the distribution of vaccines, and other considerations.

## Abbreviations

eGFR: estimated glomerular filtration rate
EHR: electronic health record
HbA_1C_: glycated hemoglobin
HR: hazard ratio
ICD: International Classification of Diseases
N3C: National COVID Cohort Collaborative
NIH: National Institutes of Health
NSF: National Science Foundation
OCS: oral corticosteroids
PCR: polymerase chain reaction
SBMI: School of Biomedical Informatics
UT: University of Texas
UTHealth: University of Texas Health Science Center
